# Sexual Segregation in Juvenile New Zealand Sea Lion Foraging Ranges: Implications for Intraspecific Competition, Population Dynamics and Conservation

**DOI:** 10.1371/journal.pone.0045389

**Published:** 2012-09-18

**Authors:** Elaine S. Leung, B. Louise Chilvers, Shinichi Nakagawa, Antoni B. Moore, Bruce C. Robertson

**Affiliations:** 1 Department of Zoology, University of Otago, Dunedin, New Zealand; 2 School of Surveying, University of Otago, Dunedin, New Zealand; 3 Department of Conservation, Aquatic & Threats Unit, Wellington, New Zealand; Université de Sherbrooke, Canada

## Abstract

Sexual segregation (sex differences in spatial organisation and resource use) is observed in a large range of taxa. Investigating causes for sexual segregation is vital for understanding population dynamics and has important conservation implications, as sex differences in foraging ecology may affect vulnerability to area-specific human activities. Although behavioural ecologists have proposed numerous hypotheses for this phenomenon, the underlying causes of sexual segregation are poorly understood. We examined the size-dimorphism and niche divergence hypotheses as potential explanations for sexual segregation in the New Zealand (NZ) sea lion (*Phocarctos hookeri*), a nationally critical, declining species impacted by trawl fisheries. We used satellite telemetry and linear mixed effects models to investigate sex differences in the foraging ranges of juvenile NZ sea lions. Male trip distances and durations were almost twice as long as female trips, with males foraging over the Auckland Island shelf and in further locations than females. Sex was the most important variable in trip distance, maximum distance travelled from study site, foraging cycle duration and percent time at sea whereas mass and age had small effects on these characteristics. Our findings support the predictions of the niche divergence hypothesis, which suggests that sexual segregation acts to decrease intraspecific resource competition. As a consequence of sexual segregation in foraging ranges, female foraging grounds had proportionally double the overlap with fisheries operations than males. This distribution exposes female juvenile NZ sea lions to a greater risk of resource competition and bycatch from fisheries than males, which can result in higher female mortality. Such sex-biased mortality could impact population dynamics, because female population decline can lead to decreased population fecundity. Thus, effective conservation and management strategies must take into account sex differences in foraging behaviour, as well as differential threat-risk to external impacts such as fisheries bycatch.

## Introduction

Sexual segregation, defined here as differential space and resource use by males and females [1], is a significant element of the life history strategy of many animals [2]. Males and females of sexually dimorphic species generally live in separate groups outside of the breeding season [2]. Investigating why the sexes differentially use habitat is an important step in understanding population processes [3]. Sexual segregation also has critical implications for population dynamics and conservation management, because sex differences in spatial dynamics can affect susceptibility to area-specific human activities [3]. For example, the foraging ranges of female wandering albatross (*Diomedea exulans*) have larger overlap with fisheries activities than males, resulting in higher bycatch mortality of females [4]. This sex-biased mortality has a drastic impact on population dynamics since female population decline can result in a reduction in the fecundity of a population [3]. Although sexual segregation is a behavioural and ecological phenomenon commonly observed in a wide variety of taxa ranging from fish to birds to mammals, the fundamental causes are poorly understood [3].

Numerous hypotheses have been advanced to explain sexual segregation [2] and two leading (non-mutually exclusive) hypotheses to elucidate sex differences in foraging behaviour outside the breeding season are the size-dimorphism and niche divergence hypotheses [5]. The size-dimorphism hypothesis proposes that species exhibiting sexual size-dimorphism will also exhibit sex differences in energetic requirements and digestive efficiencies [3]. This hypothesis states that although the larger sex has greater absolute metabolic requirements, the smaller sex requires higher-quality foods due to their relatively higher mass-specific metabolic rate and lower digestive efficiency ([6]; e.g. red deer, *Cervus elaphus* [7]; African elephants, *Loxodonta africana* [8]). The niche divergence hypothesis posits that sex differences in foraging act to reduce intraspecific competition for resources through the natural selection for different niche/resource use ([9], [10]; e.g. downy woodpeckers, *Picoides pubescens* [11]; black-capped chickadees, *Parus atricapillus* [12]). It is difficult to tease these hypotheses apart precisely because they both predict that males and females will forage in different areas. Although experimental approaches are ultimately required to separate these hypotheses (e.g. one can test if sexual segregation in foraging areas reduces inter-sexual competition by the experimental removal of one sex), such experimental interventions are logistically difficult and/or ethically undesirable. For many species, observational studies of wild animals are the only way to glean knowledge on the mechanisms that lead to sexual segregation.

The causes of sexual segregation have been predominantly studied in terrestrial vertebrates [2]. Although sexual segregation has also been observed in several marine taxa (e.g. American eel, *Anguilla rostrata* [13]; wandering albatross [4]; New Zealand fur seals, *Arctocephalus forsteri* [14]), for the most part the occurrence of this phenomenon has only been described, but the causes not investigated [3]. Both terrestrial and marine sexual segregation studies have focused mainly on breeding-age individuals. However, studies of breeding individuals can be confounded by the differential roles of each sex in reproduction and parental care, which impose different nutritional demands [5]. Breeding females and males do not face the same constraints on how long and how far they can forage since females are restricted in their foraging ranges while provisioning offspring [15]. Studying non-breeders provides an alternative route to assess sex effects because the foraging behaviour of breeders may differ considerably when not restricted by caring for young.

The New Zealand (NZ) sea lion (*Phocarctos hookeri*) provides an ideal study system to examine two of the main competing hypotheses for sexual segregation: the size-dimorphism (sex-related size differences) or niche divergence (decreasing intraspecific resource competition) hypotheses. Adult male and female NZ sea lions are thought to forage in different areas [16], [17] and this species is sexually size-dimorphic, with males being larger than females from birth [18]. Females become sexually mature at four years of age, while males are sexually mature at six years, although males do not typically breed until they are around nine years-old [19]. It is important to note this species is one of the rarest otariids (sea lions and fur seals) in the world [20] and is listed as “Vulnerable” by the International Union for Conservation of Nature [21] and as “Nationally Critical” under the NZ threat classification system [22]. The predominant anthropogenic threat to this species is a commercial trawl fishery targeting arrow squid (*Notodarus sloanii*) around the subantarctic Auckland Islands, both through mortality in bycatch and potential resource competition [23]. Therefore, understanding the mechanisms underpinning the foraging patterns of the NZ sea lion is essential to mediate the conflict between conservation efforts and commercial fisheries.

Our study aimed to answer three main questions: (1) What are the foraging ranges and at sea movements of juvenile NZ sea lions at the Auckland Islands? (2) Are the size-dimorphism and niche divergence hypotheses potential explanations for sexual segregation in the foraging ranges of NZ sea lions? Under the size-dimorphism hypothesis, although males are larger and would require more energy per unit time to meet energy requirements, females require higher quality (i.e. higher energy) prey due to the allometric relationship between metabolic requirements and body size/gut capacity [2]. Therefore, we expect sexual segregation to occur mainly due to size effects and for the larger males to forage in habitat with abundant but lower quality food, while the smaller females forage in high quality habitat [6]. From the niche divergence hypothesis, we predict sexual segregation to occur primarily due to sex effects and for males and females to forage in different areas, irrespective of prey or habitat quality, to avoid intraspecific competition for resources. And lastly, (3) Does the sexual segregation in the foraging locations of juvenile NZ sea lions result in sex-biased susceptibility to fisheries-related impacts?

## Materials and Methods

### Ethics Statement

This study was conducted with approval from the Department of Conservation (DOC) and University of Otago Animal Ethics Committees (permit numbers DOC AEC158-10 Dec. 2007, DOC AEC 200-2 Nov. 2009 and University of Otago AEC 28/10). Instrument deployments were performed under full inhalant gas anaesthesia, and all efforts were made to minimize pain and suffering.

### Capture and Deployment

**Figure 1:**
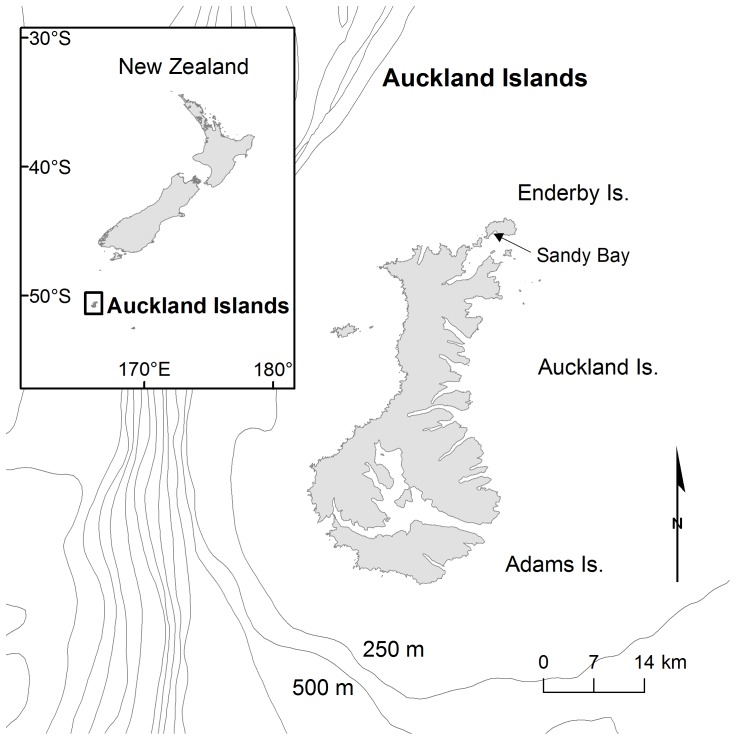
Sandy Bay study site, Enderby Island, Auckland Islands, New Zealand (50°30′S, 166°17′E).

We collected data over four austral summers from January-February, 2007–2010 at Sandy Bay, Enderby Island in the NZ subantarctic Auckland Islands (50°30′S, 166°17′E; Fig. 1). Study animals were chosen based on flipper tags that identified their age. We captured known-aged juvenile (2–3 years-old female and 2–5 years-old male) NZ sea lions using a specially designed hoop net and physically restrained them with two handlers [17]. The juveniles were anaesthetised using isoflurane delivered with oxygen to a mask via a field-portable vaporiser [24] and then strapped into a custom designed restraint frame and weighed using a 200 kg capacity scale (±0.5 kg, Salter Housewares) suspended from an aluminium tripod.

Sea lions were instrumented with satellite-linked platform transmitting terminals (Telonics 300 mW ST6, 130 mm×35 mm×15 mm, 175 g, Telonics Mesa, Arizona, U.S.A. or SPLASH, 100 mm×35 mm×35 mm, 150 g, Wildlife Computers, Redmond, Washington, U.S.A.) and also vehicle-borne high frequency (VHF; 3 cm×5 cm×2 cm, 15 g, Sirtrack, Havelock North, NZ) transmitters to facilitate recaptures. We attached the instruments to the dorsal pelage of the animal below the shoulder blades on the back midline using two-part epoxy resin. Once the instruments were securely attached, we stopped the flow of anaesthetic and the sea lion was allowed to recover. We observed each animal until it was fully conscious and had returned to the location of capture. Where possible, we recaptured the animals before the end of the field season (February 18) to retrieve instruments.

### Satellite Location Analysis

Animal positions at sea were calculated by the Argos satellite system and detailed information on the satellite location classes and number of locations received is provided in (Table S1). We filtered the satellite locations using a robust state-space model, fitted into a hierarchical Bayesian context, which can account for multiple and complex sources of data variability ([25]; see this reference for details). We ran the models using WinBUGS [26] and R [27]. The analyses were conducted hierarchically by grouping tracks from multiple individuals within the same age-sex group. To fit the model, two Markov Chain Monte Carlo (MCMC) chains were run at a four hour time step for 40 000 iterations, with a burn-in of 20 000. To reduce sample autocorrelation, every tenth point of the remaining 20 000 samples was retained for a net of 4000 MCMC samples in each chain.

After filtering satellite data with the state-space model, we defined complete foraging trips as trips with the start and end locations at Sandy Bay or within 10 km of Enderby Island following Chilvers 2008a. We restricted calculations of mean distance travelled per trip to complete foraging trips. Maximum distance travelled from Enderby Island per foraging trip was measured in ArcGIS 9.3.1 [28] as the straight-line distance from the furthest recorded point to Sandy Bay, using great circle measurement type. Foraging cycle duration was calculated by adding at sea duration with the subsequent ashore duration (except for individuals that only had one foraging trip since the subsequent ashore duration was disrupted by instrument retrieval). We used filtered locations to calculate the 50% and 95% kernel utilisation distributions (UDs) using Home Range Tools [29] for ArcGIS. We calculated fixed kernel UDs using smoothing factors calculated from the ad hoc method [30].

### Squid Trawl Fisheries

The squid fisheries operate between January-May on the Auckland Island shelf, utilising mid-water column and bottom trawls. To assess the potential sex differences in the overlap of juvenile NZ sea lion foraging grounds with the squid trawl fisheries, we mapped the 50% and 95% kernel UDs of combined fishing effort from 2007–2010 (NZ Ministry of Fisheries), along with the kernel UDs of the male and female satellite locations from 2007–2010. To quantify individual sea lion overlap with fisheries, we mapped the 50% and 95% kernel UDs of combined fishing effort from 2007–2010 (NZ Ministry of Fisheries) along with UDs of individual female and male juvenile NZ sea lions.

### Statistical Analysis

Although inter-annual variation may influence the foraging behaviour observed, small sample sizes per year precluded the differentiation of annual and individual differences. Furthermore, foraging studies on individual adult female NZ sea lions followed across several years indicate no annual variation in foraging behaviour [31]. Hence, we pooled the data across all years. We tested for mass differences between male and female juvenile NZ sea lions with t tests. Foraging behaviour was characterized by trip distance, maximum straight-line distance from the study site, foraging cycle duration and percent time spent at sea per foraging cycle. We ran multiple linear mixed effects models using foraging trip characteristics as response variables and individual animal as the random effect on repeated measures data, using the R package nlme [32]. The linear mixed effects models contained five predictor variables (sex, age, mass and the interactions sex:mass and age:mass), reduced to three predictors (sex, age and mass) after backwards stepwise selection based on Akaike Information Criterion correction for small sample size (AIC_C_) scores. Where necessary, the response variables were power or square root transformed to improve the normality of the model residuals. To improve the interpretability of regression coefficients, we centred and standardized the age and mass predictor variables [33]. Repeatability (intra-class correlation, ICC), which examines variance attributed to between individual variability, was calculated from linear mixed effects models fitted by restricted maximum likelihood [34]. Confidence intervals (CI) for repeatabilities were estimated using parametric bootstrapping [34].

To identify the relative importance of the predictor variables (sex, age, mass, sex:mass and age:mass) across the different foraging trip characteristics and to generate weighted coefficient estimates, we used a model averaging approach based on the Akaike Information Criterion correction for small sample size (AIC_C_) scores [35]. We fitted a global model in R using the maximum likelihood method. A full sub-model set was generated from the global model using the R package MuMIn [36]. Models were ranked by their AICc scores and models with Δ <2 were included in the confidence model set [35]. To determine which variables had the strongest effect on foraging trip characteristics, we averaged the models using the zero method [37], where a parameter estimate (and error) of zero are substituted into models where the parameter is absent and parameter estimates are calculated by averaging over the model set [35]. Heteroscedasticity within sex, age and mass groups were tested for each characteristic [38], but was only incorporated in the models if the heteroscedastic models had improved AICc scores. Lists of the model sets are provided in (Tables S2 and S3).

## Results

From January–February, 2007–2010, we deployed a total of 33 satellite tags on 19 female (2–3 years-old) and 14 male (2–5 years-old) juvenile NZ sea lions (Table 1). Two males (4 and 5 years-old) travelled over 500 km towards the NZ mainland shortly after instrument deployment and were not included in data analysis as these animals were considered to have dispersed [39]. Trip distance and foraging cycle duration were positively related for all juveniles (F_1, 136_ = 93.6, *P*<0.001).

**Table 1:** Age, size and number of days instruments deployed for female and male juvenile New Zealand sea lions (*Phocarctos hookeri*).

Sex	Animal id	Age	Mass (kg)	Length (cm)	Girth (cm)	No. of days deployed
F	5876	2	55.5	152	81	15
F	6111	2	56.0	138	86	21
F	6463	2	73.5	146	91	40
F	7445	2	53.0	138	83	8
F	7458	2	57.0	140	90	8
F	7610	2	54.0	140	84	23
F	8023	2	54.0	135	84	14
F	5121	3	72.0	155	90	20
F	5142	3	65.0	149	89	24
F	5857	3	71.0	141	92	14
F	5863	3	68.0	152	89	3
F	5913	3	68.0	156	95	12
F	6059	3	84.5	154	96	5
F	6130	3	68.0	153	87	19
F	6363	3	79.0	165	98	9
F	6536	3	70.0	157	93	19
F	7199	3	78.5	154	107	17
F	7458	3	73.0	153	98	11
F	7584	3	68.0	152	100	8
Mean ± SE			66.7±2.2	148.9±1.9	91.2±1.5	15.3±2.0
M	8179	2	77.5	156	91	69
M	6214	3	81.0	160	104	9
M	6218	3	76.0	155	92	41
M	6485	3	85.0	159	98	12
M	7260	3	89.0	157	111	10
M	1	4	84.0	164	101	33
M	2	4	100.0	183	106	25
M	2768	4	113.0	185	106	17
M	3257	4	93.5	166	105	33
M	3727	5	102.0	177	106	17
M	4121	5	103.5	180	108	15
M	4907	5	117.0	184	107	14
Mean ± SE			93.5±3.9	168.8±3.5	102.9±1.8	24.6±5.0

### Sex Differences

**Figure 2:**
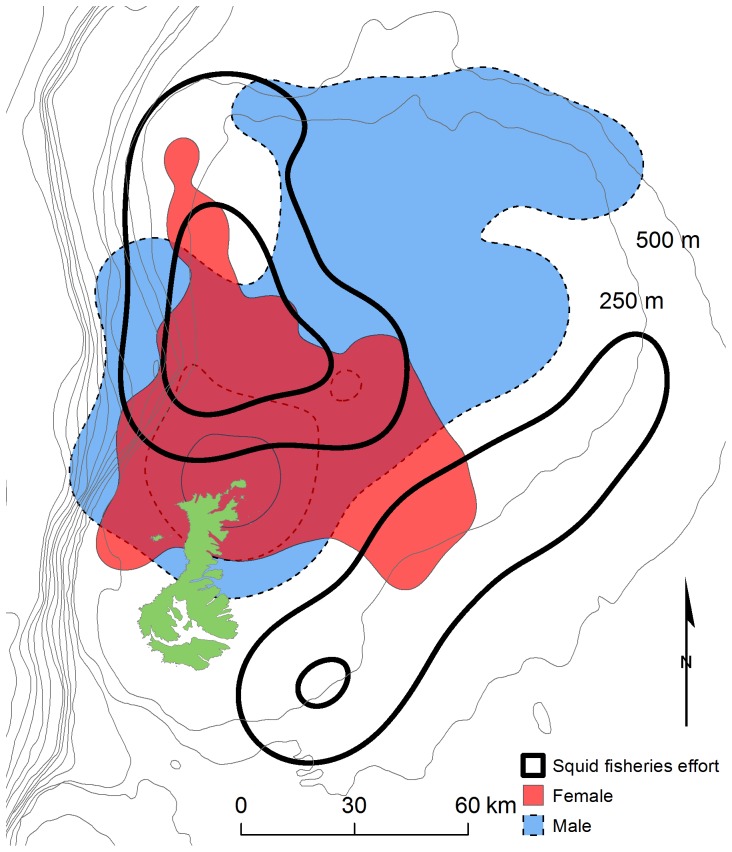
Utilisation distributions of female and male juvenile New Zealand sea lions (*Phocarctos hookeri*) and fisheries. Bathymetric contours are shown as black lines. The Auckland Island shelf is represented by the 500 m bathymetric boundary.

**Figure 3:**
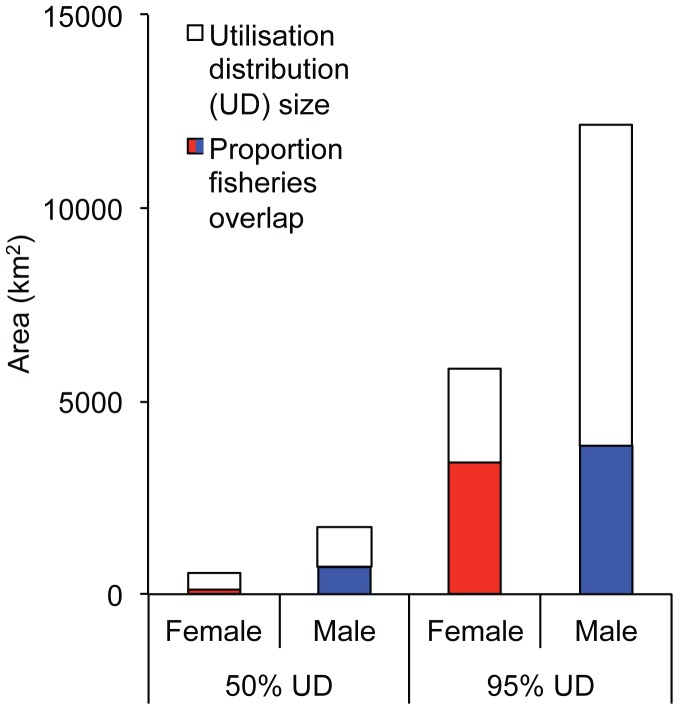
Utilisation distribution (UD) size of female and male juvenile New Zealand sea lions (*Phocarctos hookeri*). The proportion of UD overlap with squid trawl fisheries operations is indicated in red or blue.

Male juvenile NZ sea lions were significantly heavier than females (Table 1; t = −6.457, df = 29, *P*<0.001). Male trip distances and maximum distance travelled from the study site were almost twice as long as female distances (Table 2; Fig. 2). Female juvenile NZ sea lions foraged mainly within 50 km of the Auckland Islands, while males foraged closer to the 500 m bathymetric boundary of the Auckland Island shelf, over 100 km from the Auckland Islands (Figs. 2 and S1). The 50% kernel UDs of males were over three times larger than females (1742 km^2^ vs. 523 km^2^), while the 95% kernel UDs of males were over two times larger than females (12149 km^2^ vs. 5836 km^2^; Fig. 3). Male juvenile NZ sea lion foraging cycle durations were over 1.5 times longer than female durations and males also spent a larger proportion of time at sea (Table 2).

**Table 2:** Foraging trip characteristics for female and male juvenile New Zealand sea lions (*Phocarctos hookeri*).

			Foraging cycle duration (h)		Time at sea (%)		*Trip distance (km)		§Max distance (km)		
Sex	Animal id	No. of foraging trips	Mean	SD	Mean	SD	Mean	SD	Mean	SD	Max
F	5876	4	69.7	47.2	58.5	46.2	72.4	38.4	23.9	4.6	30.8
F	6111	6	81.2	9.0	61.0	20.1	116.9	16.9	47.2	8.6	58.4
F	6463	8	149.5	62.3	77.0	22.5	304.0	113.8	62.5	19.8	91.8
F	7445	4	40.6	4.7	72.2	19.1	91.4	23.2	29.6	1.9	30.9
F	7458	3	49.6	56.8	65.6	31.2	75.1	27.7	21.0	7.2	29.1
F	7610	7	76.5	19.8	57.4	5.2	136.2	31.6	48.5	8.7	56.7
F	8023	5	50.7	22.3	65.6	25.1	107.7	58.4	41.5	20.3	53.3
F	5121	5	79.8	17.2	82.2	11.1	165.2	16.5	62.5	9.6	79.2
F	5142	9	61.8	23.1	53.7	21.3	60.0	24.9	23.5	11.5	54.0
F	5857	6	36.4	12.4	74.0	21.1	74.9	40.8	30.1	15.6	50.0
F	5863	1	28.8		N/A	N/A	68.3		29.9	NA	29.9
F	5913	3	71.3	9.5	90.5	13.4	184.3	53.8	73.2	27.4	103.1
F	6059	1	93.5		N/A	N/A	100.5		18.2	NA	18.2
F	6130	6	57.7	7.7	74.8	13.7	136.9	20.9	51.8	11.3	62.6
F	6363	3	52.3	18.4	82.4	18.2	94.8	32.9	32.2	7.8	36.9
F	6536	7	54.5	17.6	67.5	16.8	80.5	19.3	32.0	9.7	51.8
F	7199	13	26.5	12.5	40.7	22.5	32.4	20.8	14.1	7.9	31.5
F	7458	4	62.9	18.8	56.5	30.4	105.1	36.4	41.3	15.9	61.1
F	7584	3	49.9	23.4	74.8	24.9	104.2	30.0	36.4	1.5	37.5
Mean ± SE		5±1	56.9±6.2		67.4±3.0		98.7±12.3		35.8±3.8		50.9±5.2
M	8179	16	102.3	33.1	79.8	14.5	245.2	93.7	81.4	38.2	138.6
M	6214	2	138.4	0.9	94.1	8.3	242.5	44.8	87.0	1.6	88.1
M	6218	12	79.8	32.8	65.2	18.3	129.6	35.3	38.4	6.9	49.9
M	6485	8	27.4	9.9	64.2	8.1	81.4	35.8	33.7	2.9	37.1
M	7260	3	59.7	16.2	73.3	10.2	128.9	38.7	46.1	4.2	50.2
M	1	6	128.7	27.8	74.3	14.1	308.0	55.1	97.2	6.3	109.1
M	2	4	142.0	61.9	65.7	29.1	318.8	179.3	102.3	48.9	132.1
M	2768	4	93.2	26.7	73.8	18.0	186.2	45.9	63.4	11.4	74.3
M	3257	6	127.5	55.7	54.2	25.8	178.3	72.7	76.6	31.0	93.2
M	3727	4	96.8	37.6	69.4	23.8	234.6	16.9	103.4	5.4	109.4
M	4121	2	140.2	29.8	93.6	9.0	371.5	18.8	133.1	8.0	138.7
M	4907	4	77.3	40.2	59.3	27.9	102.4	73.6	39.6	34.9	91.9
Mean ± SE		6±1	91.0±10.6		70.7±3.3		183.9±25.4		68.4±7.5		92.7±10.1

* Trip distances represent complete foraging trips.

§ Max distances represent maximum straight-line distance from the furthest recorded point to the study site.

Sex had the most important effect on trip distance, maximum distance from study site, foraging cycle duration and percent time at sea (Tables 3, S2 and S3). The effect of sex on trip distance, maximum distance from study site and foraging cycle duration was large as the confidence interval for sex for these characteristics did not include zero (Table 3). Both mass and age had small effects on these characteristics (Table 3). The ICC coefficients for the random effects term (animal identity) explained most of the variation and were significantly repeatable for trip distance (ICC = 0.646, 95% CI = 0.471 to 0.768, *P*<0.001), maximum distance from study site (ICC = 0.654, 95% CI = 0.474 to 0.766, *P*<0.001) and foraging cycle duration (ICC = 0.545, 95% CI = 0.364 to 0.687, *P*<0.001). Significant repeatability estimates indicate male juvenile NZ sea lions consistently travelled further distances for longer periods than females.

**Table 3:** Summary results of linear mixed effects models run on juvenile New Zealand sea lion (*Phocarctos hookeri*) foraging trip characteristics: effects of each variable on trip distance, maximum distance from study site, foraging cycle duration and percent time spent at sea.

Trip characteristic	Variable	Estimate*	SE	Lower 95% CI	Upper 95% CI	Relative importance§	ICC
Trip distance (km; power transformed)	(Intercept)	20.100	0.670	18.800	21.400		0.646
	Sex (Male)	3.530	1.270	1.050	6.010	**1.00**	
	Age	0.009	0.280	−0.540	0.558	0.20	
	Mass	0.001	0.330	−0.646	0.649	0.20	
Max distance from study site (km; square root transformed)	(Intercept)	6.020	0.355	5.320	6.710		0.654
	Sex (Male)	2.170	0.703	0.795	3.550	**1.00**	
	Age	0.080	0.217	−0.345	0.505	0.28	
	Mass	0.003	0.167	−0.324	0.331	0.18	
Trip duration (h; power transformed)	(Intercept)	3.710	0.136	3.440	3.980		0.545
	Sex (Male)	0.577	0.255	0.077	1.080	**1.00**	
	Age	0.007	0.057	−0.105	0.118	0.21	
	Mass	0.012	0.071	−0.127	0.151	0.22	
Time spent at sea (%)	(Intercept)	0.686	0.025	0.636	0.735		0.158
	Sex (Male)	0.007	0.025	−0.042	0.056	0.21	
	Age	0.000	0.009	−0.017	0.018	0.16	
	Mass	0.002	0.010	−0.017	0.021	0.18	

* Effect sizes have been standardised following Schielzeth (2010).

§ Relative importance values in bold indicate the confidence intervals for these parameter estimates do not include zero, indicating these predictor variables have a strong effect on foraging behaviour.

CI, confidence interval; ICC, intra-class correlation.

### Sea Lion-fishery Overlap

**Figure 4:**
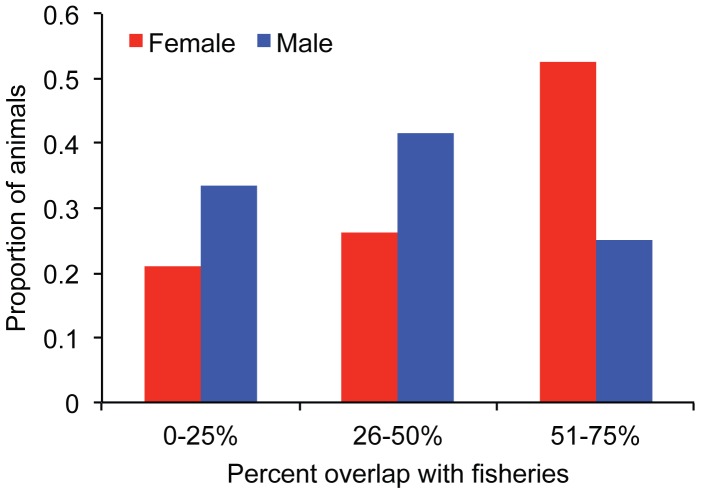
Proportion of female and male juvenile New Zealand sea lions (*Phocarctos hookeri*) with fisheries overlap. Individuals had 0–75% of their 95% kernel utilisation distribution overlap with squid trawl fisheries operations.

Juvenile NZ sea lions had temporal and spatial overlap with squid fisheries. Female juvenile NZ sea lion foraging ranges had minor overlap with the squid fishery in the southeast while male foraging grounds had no overlap with fisheries in this area (Fig. 2). Both female and male juvenile foraging ranges had large overlap with the squid trawl fisheries north of the Auckland Islands (Fig. 2). The 95% kernel UD of females and males had a similar total amount of overlap with squid trawl fisheries (3395 km^2^ vs. 3844 km^2^). As a percentage of the 95% kernel UD area, female foraging grounds had proportionally almost double the amount of overlap with squid trawl fisheries operations than males (Fig. 3). Fifty-eight percent of the females’ 95% kernel UD overlapped with squid trawl fisheries vs. 32% for males. When individual habitat use in relation to fisheries overlap is considered (Figs. S2–S3), 10 out of 19 females and 3 out of 12 males had >50% of their 95% kernel UDs overlap with fisheries (Fig. 4). None of the juvenile NZ sea lions had >75% of their 95% kernel UDs overlap with fisheries.

## Discussion

Male juvenile NZ sea lions foraged over the Auckland Island shelf and in further locations than females; this would likely result in decreasing intraspecific resource competition. Our study provides greater support for the niche divergence hypothesis than the size-dimorphism hypothesis as a potential explanation for the observed sex differences in the foraging ranges of juvenile NZ sea lions. Sexual segregation in the foraging behaviour of juvenile NZ sea lions were mainly due to sex effects, rather than mass or age influences. However, sex differences in the foraging locations of juvenile NZ sea lions has conservation implications; the majority of female foraging grounds overlap with fisheries operations and consequently, female juvenile NZ sea lions are more susceptible than males to fisheries impacts through bycatch and resource competition. This sex-biased threat-risk has major repercussions for population dynamics since female population decline can lead to decreases in population fecundity.

### Niche Divergence Hypothesis

As female juvenile NZ sea lions utilize different foraging space horizontally (Fig. 2) and also vertically (i.e. different depth ranges; E. Leung, unpublished data) from male juveniles, this would result in niche specialization that likely minimizes intraspecific competition for prey resources. Sex played a more important role than mass and age in influencing all of the foraging trip characteristics (Table 2). It is difficult to separately delineate the effects of sex, mass and age on NZ sea lion foraging behaviour, because these variables are all interrelated, with males being larger than females of the same age-class and older animals being larger than younger individuals. However, the low importance of mass and age indicates sexual segregation in juvenile NZ sea lions is largely due to factors intrinsic to each sex as opposed to size or age-related effects associated with sexual size-dimorphism. Similarly, sex rather than body size, accounted for the sex differences in diving behaviour of sexually dimorphic grey seals (*Halichoerus grypus* [5]).

Female and male juvenile NZ sea lions also forage in different areas from adult females [17] and adult males [16], further supporting the niche divergence hypothesis. The reduction of intraspecific competition through inter-sexual niche partitioning has been reported in a wide range of sexually dimorphic taxa (e.g. lizards, *Anolis polylepis* [40]; pythons, *Morelia spilota imbricata* [41]; cormorants, *Phalacrocorax albiventer* [42]; wandering albatross [4]; NZ fur seals [14]; southern elephant seals, *Mirounga leonina* [43]). The experimental removal of male birds from feeding territories resulted in the niche shift of females to habitat niches usually used only by males, thus providing additional evidence for the role of intraspecific competition in influencing the differential use of niches by each sex (e.g. downy woodpeckers [11]; black-capped chickadees [12]). However, the mechanisms that lead to sexual segregation may not necessarily be the same factors that maintain it. It is difficult to pinpoint a single hypothesis as the sole cause of sexual segregation because it is simultaneously influenced by numerous, often interrelated, environmental and behavioural factors [1]. Although this study provides more support for the niche divergence hypothesis than the size-dimorphism hypothesis, it is possible that decreasing intraspecific competition acts to maintain sexual segregation, rather than being the causal mechanism.

### Size-dimorphism Hypothesis

The size-dimorphism hypothesis predicts that females require higher-quality foods due to their relatively higher mass-specific metabolic rate and lower digestive efficiency and thus will forage on higher energy prey in higher quality habitat than males [6]. Although the juvenile male NZ sea lions tagged in this study were significantly heavier than juvenile females (male mean mass 1.4 times heavier than female mean mass), mass had little influence on juvenile NZ sea lion foraging trip characteristics (Table 3). However, it is possible that mass effects on foraging behaviour may be stronger in sexually mature individuals where sexual size dimorphism is more pronounced (e.g. adult male NZ sea lions are 2.5 times heavier than adult females [44]). Nevertheless, contrary to predictions of the size-dimorphism hypothesis, adult male and female NZ sea lions consume the same prey species and sex differences in prey quality are not evident from diet studies on this species [45]. It is important to note that juvenile NZ sea lions do not completely sexually segregate since there is overlap between the male and female kernel UDs because males travel through female foraging areas to reach their farther foraging grounds (Fig. 2). As we did not measure incidences of food intake, we do not know whether males feed in the same areas as females on their way to their farther foraging locations. However, male juvenile NZ sea lions spend more time at sea in transit (60%) than diving, indicating males likely forage in further locations (E. Leung, unpublished data).

Our results add to the mounting evidence against the size-dimorphism hypothesis [1] and may assist in reducing the number of possible hypotheses for the occurrence of sexual segregation. A contentious prediction of the size-dimorphism hypothesis is the competitive exclusion of the larger sex from high quality habitats by the greater foraging efficiency of the smaller sex [3]. This phenomenon is rare in marine vertebrates and indeed the competitive exclusion of the smaller sex by the larger sex appears more common [3]. Furthermore, the size-dimorphism hypothesis has been deemed untenable in ungulates [1] and contrary to the predictions of this hypothesis, male (the larger sex) wandering albatross forage in more profitable habitats than females and also forage more efficiently [46]. Lastly, sexual size-dimorphism cannot be the only explanation for sexual segregation as many monomorphic species also display sexual segregation [2].

### Conservation Implications

Juvenile NZ sea lions overlap temporally and spatially (horizontally and vertically) with squid trawl fisheries. From 2007–2010, the squid fisheries operated at depths 98–338 m (NZ Ministry of Fisheries). Sixty-one percent of tagged female and 47% of male juvenile NZ sea lions dives were within the depth range targeted by squid trawl fisheries (E. Leung, unpublished data). Based on the assumption that male and female NZ sea lions behave similarly in fishing areas (e.g. one sex does not avoid trawl nets more than the other sex), a consequence of the sexual segregation in the foraging ranges of juvenile NZ sea lions is the higher risk of resource competition and bycatch of females in the squid fishery. Juvenile female NZ sea lion foraging grounds had proportionally double the overlap with fisheries operations than males (Fig. 3). Of the tagged individuals, over half of the females had greater than 50% of their foraging range overlap with fisheries, compared to only one-quarter of the males (Fig. 4), suggesting that the stronger overlap of females with fisheries is not due to a few individuals. Indeed, the majority of NZ sea lions caught annually in the squid fisheries are adult females [47] and juvenile females [48], [49]. This sex-biased threat-risk needs to be accounted for in the conservation and management of this nationally critical, declining species.

Sex-biased mortality of females in fishery operations have been reported for several species (e.g. wandering albatross [4] and numerous fish species [50]). Female-biased mortality has a larger impact on population dynamics in polygynous species than male-biased mortality, because male mating success is variable, whereas most females will either be pregnant or provisioning offspring for the majority of their reproductive years [3]. Therefore, female population decline can lead to a reduction in the fecundity of a population [3]. The widespread occurrence of sexual segregation throughout many taxa and the consequent potential for sex-biased threat risk advocates for the separate consideration of the sexes in population models and management plans [3].

### Conclusions

Although it is likely that numerous factors influence the sexual segregation observed in the NZ sea lion, our results give greater support for the niche divergence than the size-dimorphism hypothesis. Examining the underlying mechanisms that result in sexual segregation allows us to understand population dynamic processes and patterns [3]. Sexual segregation in the foraging ranges of juvenile NZ sea lions would result in niche specialization that likely minimizes intraspecific competition for prey resources. However, this sexual segregation has strong conservation and management implications because the foraging grounds of female juvenile NZ sea lions have larger overlap with fisheries activities than males, resulting in females being at higher risk of fisheries-related threats (i.e. bycatch and resource competition). This sex-biased threat-risk emphasizes the importance of investigating the spatiotemporal dynamics of the sexes separately in species threatened by area-specific human activities. Such incidences of sex-biased mortality may lead to extirpation of a species, especially where populations are small or female numbers are low [2].

## Supporting Information

Figure S1 **Satellite locations of female and male juvenile New Zealand sea lions (** ***Phocarctos hookeri*** **) from 2007–2010.** The Auckland Islands are represented in light grey. Bathymetric contours are shown as black lines. The Auckland Island shelf is represented by the 500 m bathymetric boundary.
(TIF)

Figure S2 **Utilisation distributions (UD) of individual female juvenile New Zealand sea lions (** ***Phocarctos hookeri*** **) and squid trawl fisheries.** Individuals with: A) 0–25%, B) 26–50% and C) 51–75% of their 95% UD overlapping with fisheries. Note panel C is represented by two figures of five individual females per map.
(TIF)

Figure S3 **Utilisation distributions (UD) of individual male juvenile New Zealand sea lions (** ***Phocarctos hookeri*** **) and squid trawl fisheries.** Individuals with: A) 0–25%, B) 26–50% and C) 51–75% of their 95% UD overlapping with fisheries.
(TIF)

Table S1 **Satellite location data for female and male juvenile New Zealand sea lions (** ***Phocarctos hookeri*** **).**
(DOC)

Table S2 **Results of linear mixed effects models run on juvenile New Zealand sea lion (** ***Phocarctos hookeri*** **) foraging trip characteristics: trip distance and maximum distance from study site.**
(DOC)

Table S3 **Results of linear mixed effects models run on juvenile New Zealand sea lion (** ***Phocarctos hookeri*** **) foraging trip characteristics: foraging cycle duration and percent time spent at sea.**
(DOC)
