# Supplementary figures and images for: Sexual Segregation in Juvenile New Zealand Sea Lion Foraging Ranges: Implications for Intraspecific Competition, Population Dynamics and Conservation
Source: PLoS One. 2012 Sep 18;7(9):e45389. doi: 10.1371/journal.pone.0045389 (PMC3445520; doi:10.1371/journal.pone.0045389)

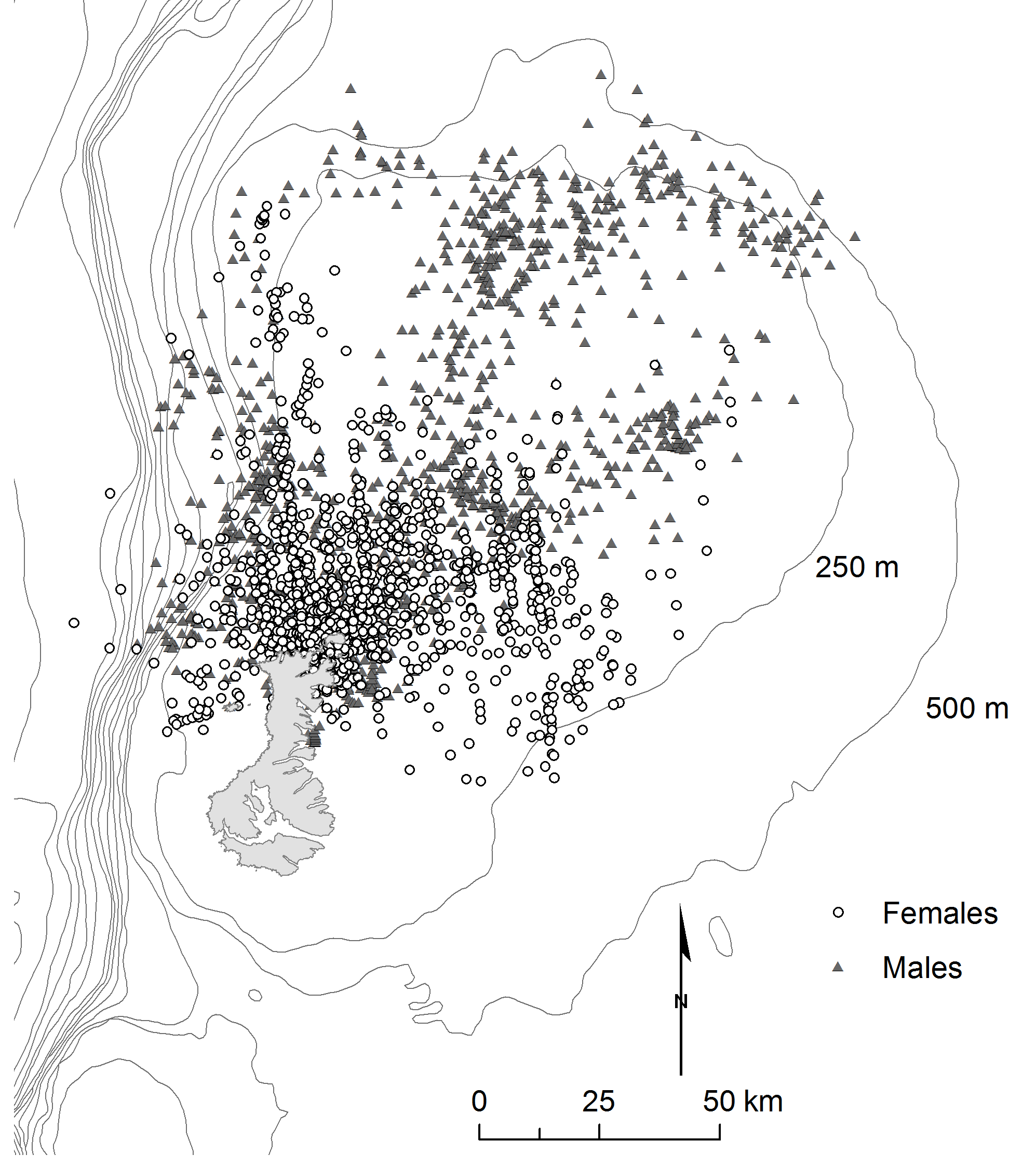

Supplement: Figure S1 — Satellite locations of female and male juvenile New Zealand sea lions ( Phocarctos hookeri ) from 2007–2010. The Auckland Islands are represented in light grey. Bathymetric contours are shown as black lines. The Auckland Island shelf is represented by the 500 m bathymetric boundary. (TIF) [file pone.0045389.s001.tif]

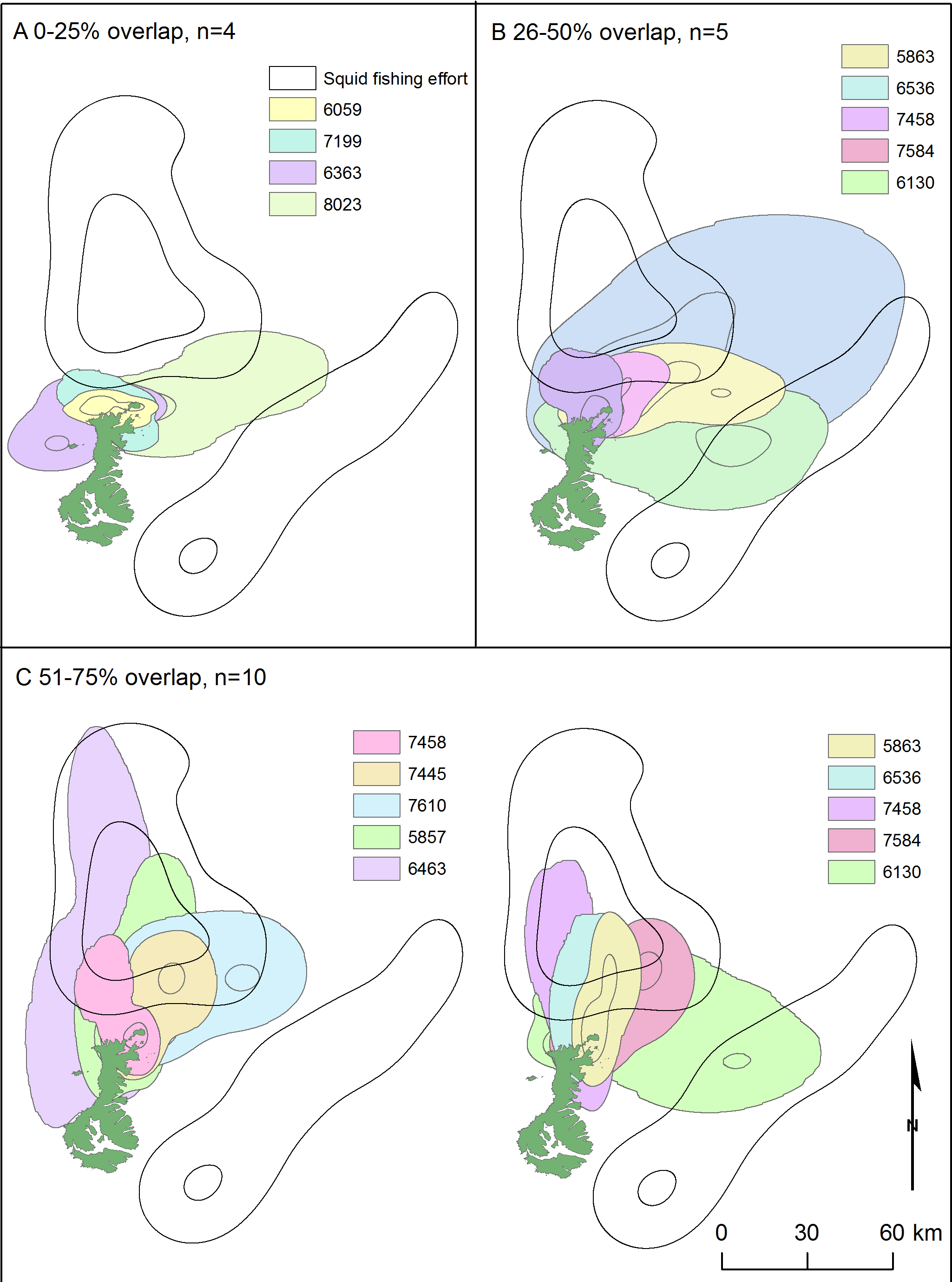

Supplement: Figure S2 — Utilisation distributions (UD) of individual female juvenile New Zealand sea lions ( Phocarctos hookeri ) and squid trawl fisheries. Individuals with: A) 0–25%, B) 26–50% and C) 51–75% of their 95% UD overlapping with fisheries. Note panel C is represented by two figures of five individual females per map. (TIF) [file pone.0045389.s002.tif]

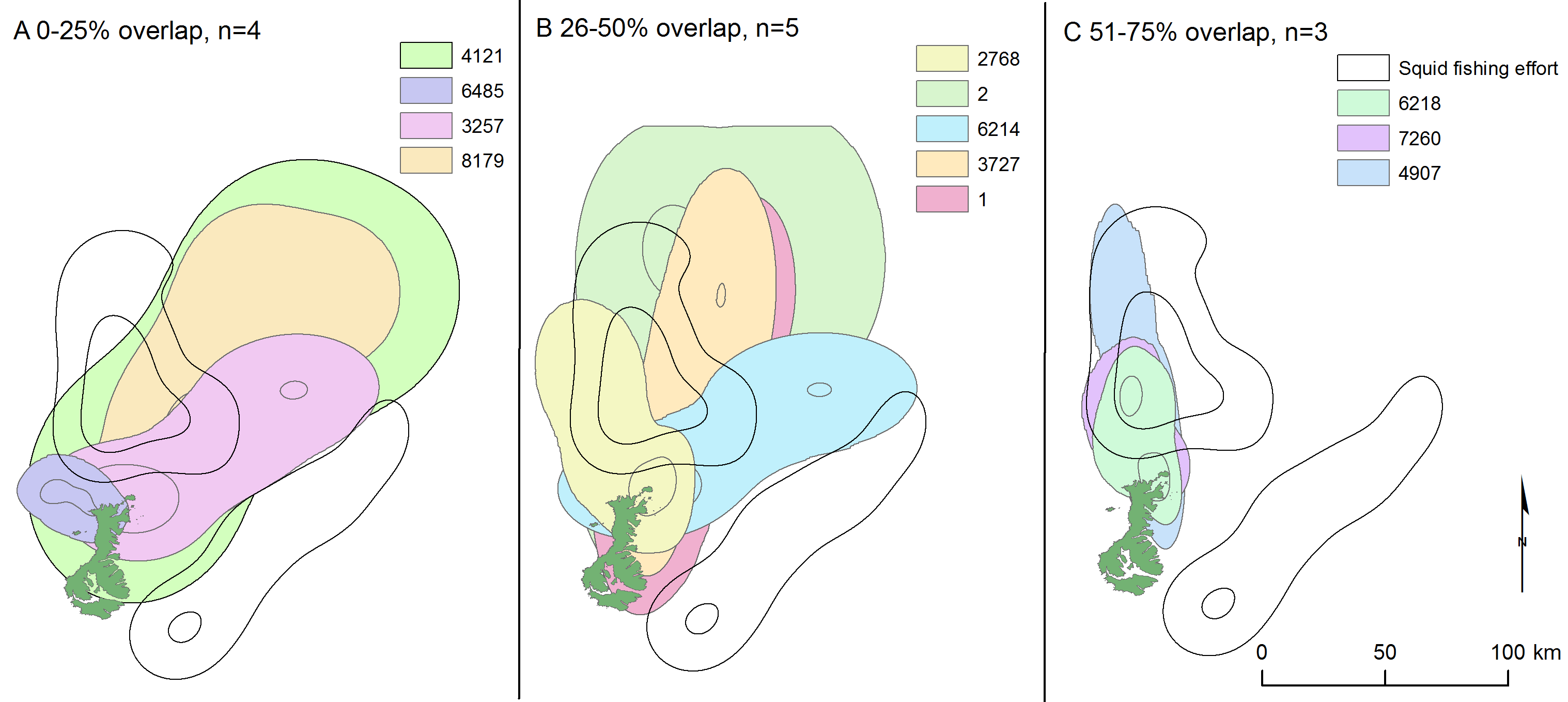

Supplement: Figure S3 — Utilisation distributions (UD) of individual male juvenile New Zealand sea lions ( Phocarctos hookeri ) and squid trawl fisheries. Individuals with: A) 0–25%, B) 26–50% and C) 51–75% of their 95% UD overlapping with fisheries. (TIF) [file pone.0045389.s003.tif]
