# Supplementary material for: Sexual Segregation in Juvenile New Zealand Sea Lion Foraging Ranges: Implications for Intraspecific Competition, Population Dynamics and Conservation
Source: PLoS One. 2012 Sep 18;7(9):e45389. doi: 10.1371/journal.pone.0045389 (PMC3445520; doi:10.1371/journal.pone.0045389)
Supplement: Table S1 — Satellite location data for female and male juvenile New Zealand sea lions ( Phocarctos hookeri ). (DOC) [file pone.0045389.s004.doc]

**Table S1. Satellite location data for female and male juvenile New Zealand sea lions (*Phocarctos hookeri*).**

|  |  |  |  | Proportion of satellite locations in location class | | | | | | |
| --- | --- | --- | --- | --- | --- | --- | --- | --- | --- | --- |
| Sex | Animal id | No. of days deployed | No. of satellite locations before filtering | 3 | 2 | 1 | 0 | A | B | Z |
| F | 5876 | 15 | 122 | 8 | 11 | 19 | 15 | 20 | 22 | 5 |
| F | 6111 | 21 | 258 | 4 | 4 | 13 | 6 | 23 | 44 | 6 |
| F | 6463 | 40 | 939 | 1 | 2 | 7 | 28 | 27 | 33 | 1 |
| F | 7445 | 8 | 149 | 11 | 15 | 22 | 12 | 15 | 24 | 0 |
| F | 7458 | 8 | 105 | 19 | 26 | 22 | 9 | 10 | 15 | 0 |
| F | 7610 | 23 | 316 | 2 | 12 | 22 | 18 | 21 | 25 | 0 |
| F | 8023 | 14 | 206 | 10 | 21 | 18 | 8 | 21 | 21 | 0 |
| F | 5121 | 20 | 265 | 5 | 9 | 11 | 9 | 20 | 45 | 2 |
| F | 5142 | 24 | 221 | 5 | 11 | 14 | 10 | 13 | 44 | 4 |
| F | 5857 | 14 | 183 | 10 | 9 | 35 | 11 | 15 | 20 | 0 |
| F | 5863 | 3 | 30 | 3 | 7 | 13 | 23 | 20 | 33 | 0 |
| F | 5913 | 12 | 217 | 4 | 6 | 17 | 21 | 26 | 26 | 0 |
| F | 6059 | 5 | 42 | 2 | 2 | 14 | 31 | 14 | 36 | 0 |
| F | 6130 | 19 | 249 | 8 | 15 | 20 | 10 | 18 | 29 | 0 |
| F | 6363 | 9 | 135 | 10 | 13 | 27 | 11 | 20 | 19 | 0 |
| F | 6536 | 19 | 235 | 11 | 14 | 23 | 12 | 23 | 15 | 0 |
| F | 7199 | 17 | 209 | 10 | 19 | 20 | 11 | 21 | 19 | 0 |
| F | 7458 | 11 | 201 | 6 | 11 | 32 | 20 | 12 | 17 | 1 |
| F | 7584 | 8 | 143 | 3 | 15 | 24 | 19 | 19 | 20 | 0 |
| M | 8179 | 69 | 1157 | 6 | 11 | 18 | 9 | 25 | 30 | 0 |
| M | 6214 | 9 | 139 | 4 | 6 | 17 | 16 | 21 | 36 | 1 |
| M | 6218 | 41 | 570 | 4 | 6 | 21 | 20 | 24 | 26 | 1 |
| M | 6485 | 12 | 208 | 1 | 4 | 15 | 27 | 25 | 27 | 0 |
| M | 7260 | 10 | 247 | 13 | 22 | 21 | 11 | 18 | 15 | 0 |
| M | 1 | 33 | 496 | 0 | 2 | 13 | 40 | 14 | 24 | 6 |
| M | 2 | 25 | 709 | 1 | 5 | 14 | 46 | 13 | 19 | 2 |
| M | 2768 | 17 | 343 | 2 | 3 | 10 | 58 | 8 | 13 | 7 |
| M | 3257 | 33 | 250 | 0 | 2 | 9 | 50 | 15 | 20 | 4 |
| M | 3727 | 17 | 316 | 5 | 7 | 12 | 34 | 17 | 25 | 0 |
| M | 4121 | 15 | 216 | 9 | 17 | 36 | 16 | 14 | 8 | 0 |
| M | 4907 | 14 | 138 | 8 | 5 | 16 | 17 | 25 | 28 | 1 |
