# Supplementary material for: Sexual Segregation in Juvenile New Zealand Sea Lion Foraging Ranges: Implications for Intraspecific Competition, Population Dynamics and Conservation
Source: PLoS One. 2012 Sep 18;7(9):e45389. doi: 10.1371/journal.pone.0045389 (PMC3445520; doi:10.1371/journal.pone.0045389)
Supplement: Table S2 — Results of linear mixed effects models run on juvenile New Zealand sea lion ( Phocarctos hookeri ) foraging trip characteristics: trip distance and maximum distance from study site. (DOC) [file pone.0045389.s005.doc]

**Table S2. Results of linear mixed effects models run on juvenile New Zealand sea lion (*Phocarctos hookeri*) foraging trip characteristics: trip distance and maximum distance from study site.**

| Trip characteristic | Model | Intercept | Sex | Age | Mass | Sex:mass | Age:mass | k | AICc | ΔAICc | ωAICc |
| --- | --- | --- | --- | --- | --- | --- | --- | --- | --- | --- | --- |
| Trip distance (km; power transformed) | 2 | 20.086 | 3.540 |  |  |  |  | 5 | 815.866 | 0.000 | 0.430 |
|  | 5 | 20.103 | 3.483 | 0.044 |  |  |  | 6 | 817.860 | 1.994 | 0.159 |
|  | 6 | 20.090 | 3.529 |  | 0.006 |  |  | 6 | 817.866 | 2.000 | 0.158 |
|  | 8 | 20.068 | 3.561 | 0.103 | -0.090 |  |  | 7 | 819.854 | 3.988 | 0.059 |
|  | 9 | 20.085 | 3.527 |  | -0.002 | 0.015 |  | 7 | 819.866 | 4.000 | 0.058 |
|  | 4 | 21.397 |  |  | 1.200 |  |  | 5 | 820.555 | 4.690 | 0.041 |
|  | 12 | 20.019 | 3.552 | 0.063 | -0.097 |  | 0.058 | 8 | 821.831 | 5.965 | 0.022 |
|  | 11 | 20.074 | 3.564 | 0.106 | -0.082 | -0.017 |  | 8 | 821.854 | 5.988 | 0.022 |
|  | 7 | 21.421 |  | -0.251 | 1.406 |  |  | 6 | 822.493 | 6.627 | 0.016 |
|  | 3 | 21.303 |  | 1.030 |  |  |  | 5 | 822.575 | 6.709 | 0.015 |
|  | 13 | 20.130 | 3.688 | 0.038 | 0.268 | -0.923 | 0.274 | 9 | 823.736 | 7.871 | 0.008 |
|  | 1 | 21.424 |  |  |  |  |  | 4 | 824.337 | 8.471 | 0.006 |
|  | 10 | 21.346 |  | -0.308 | 1.392 |  | 0.082 | 7 | 824.452 | 8.586 | 0.006 |
| Max distance from study site (km; square root transformed) | 2 | 5.987 | 2.284 |  |  |  |  | 5 | 616.513 | 0.000 | 0.346 |
|  | 5 | 6.094 | 1.907 | 0.287 |  |  |  | 6 | 617.679 | 1.166 | 0.193 |
|  | 6 | 5.998 | 2.254 |  | 0.017 |  |  | 6 | 618.511 | 1.998 | 0.127 |
|  | 8 | 5.859 | 2.451 | 0.707 | -0.629 |  |  | 7 | 618.523 | 2.010 | 0.127 |
|  | 9 | 5.868 | 2.183 |  | -0.191 | 0.387 |  | 7 | 620.237 | 3.724 | 0.054 |
|  | 11 | 5.803 | 2.410 | 0.681 | -0.703 | 0.182 |  | 8 | 620.463 | 3.950 | 0.048 |
|  | 12 | 5.840 | 2.445 | 0.691 | -0.631 |  | 0.023 | 8 | 620.509 | 3.996 | 0.047 |
|  | 13 | 5.787 | 2.369 | 0.700 | -0.809 | 0.465 | -0.086 | 9 | 622.421 | 5.908 | 0.018 |
|  | 3 | 6.726 |  | 0.832 |  |  |  | 5 | 622.678 | 6.165 | 0.016 |
|  | 4 | 6.806 |  |  | 0.773 |  |  | 5 | 623.034 | 6.521 | 0.013 |
|  | 7 | 6.757 |  | 0.500 | 0.362 |  |  | 6 | 624.203 | 7.690 | 0.007 |
|  | 10 | 6.709 |  | 0.465 | 0.351 |  | 0.052 | 7 | 626.148 | 9.635 | 0.003 |
|  | 1 | 6.831 |  |  |  |  |  | 4 | 628.806 | 12.293 | 0.001 |
