# Supplementary material for: Sexual Segregation in Juvenile New Zealand Sea Lion Foraging Ranges: Implications for Intraspecific Competition, Population Dynamics and Conservation
Source: PLoS One. 2012 Sep 18;7(9):e45389. doi: 10.1371/journal.pone.0045389 (PMC3445520; doi:10.1371/journal.pone.0045389)
Supplement: Table S3 — Results of linear mixed effects models run on juvenile New Zealand sea lion ( Phocarctos hookeri ) foraging trip characteristics: foraging cycle duration and percent time spent at sea. (DOC) [file pone.0045389.s006.doc]

**Table S3. Results of linear mixed effects models run on juvenile New Zealand sea lion (*Phocarctos hookeri*) foraging trip characteristics:** foraging cycle duration **and percent time spent at sea.**

| Trip characteristic | Model | Intercept | Sex | Age | Mass | Sex:mass | Age:mass | k | AICc | ΔAICc | ωAICc |
| --- | --- | --- | --- | --- | --- | --- | --- | --- | --- | --- | --- |
| Foraging cycle duration (h; power transformed) | 2 | 3.701 | 0.607 |  |  |  |  | 4 | 313.269 | 0.000 | 0.353 |
|  | 6 | 3.736 | 0.509 |  | 0.056 |  |  | 5 | 315.100 | 1.831 | 0.141 |
|  | 5 | 3.713 | 0.565 | 0.032 |  |  |  | 5 | 315.192 | 1.923 | 0.135 |
|  | 4 | 3.925 |  |  | 0.227 |  |  | 4 | 315.655 | 2.385 | 0.107 |
|  | 9 | 3.725 | 0.505 |  | 0.038 | 0.031 |  | 6 | 317.088 | 3.819 | 0.052 |
|  | 8 | 3.739 | 0.505 | -0.013 | 0.068 |  |  | 6 | 317.095 | 3.826 | 0.052 |
|  | 7 | 3.931 |  | -0.064 | 0.280 |  |  | 5 | 317.540 | 4.270 | 0.042 |
|  | 3 | 3.911 |  | 0.191 |  |  |  | 4 | 317.866 | 4.596 | 0.035 |
|  | 12 | 3.707 | 0.504 | -0.039 | 0.060 |  | 0.035 | 7 | 318.874 | 5.604 | 0.021 |
|  | 11 | 3.726 | 0.499 | -0.018 | 0.052 | 0.036 |  | 7 | 319.079 | 5.810 | 0.019 |
|  | 10 | 3.898 |  | -0.090 | 0.272 |  | 0.036 | 6 | 319.328 | 6.059 | 0.017 |
|  | 1 | 3.940 |  |  |  |  |  | 3 | 319.498 | 6.229 | 0.016 |
|  | 13 | 3.750 | 0.552 | -0.051 | 0.200 | -0.341 | 0.114 | 8 | 320.529 | 7.259 | 0.009 |
| Time at sea (%) | 1 | 0.689 |  |  |  |  |  | 4 | -33.776 | 0.000 | 0.317 |
|  | 2 | 0.674 | 0.033 |  |  |  |  | 5 | -32.298 | 1.478 | 0.151 |
|  | 4 | 0.688 |  |  | 0.010 |  |  | 5 | -32.017 | 1.759 | 0.131 |
|  | 3 | 0.689 |  | 0.003 |  |  |  | 5 | -31.795 | 1.981 | 0.118 |
|  | 5 | 0.670 | 0.044 | -0.009 |  |  |  | 6 | -30.429 | 3.348 | 0.059 |
|  | 7 | 0.689 |  | -0.022 | 0.028 |  |  | 6 | -30.318 | 3.458 | 0.056 |
|  | 6 | 0.672 | 0.038 |  | -0.003 |  |  | 6 | -30.306 | 3.470 | 0.056 |
|  | 9 | 0.697 | 0.035 |  | 0.036 | -0.060 |  | 7 | -29.185 | 4.592 | 0.032 |
|  | 10 | 0.701 |  | -0.012 | 0.031 |  | -0.014 | 7 | -28.953 | 4.824 | 0.028 |
|  | 8 | 0.675 | 0.031 | -0.018 | 0.014 |  |  | 7 | -28.518 | 5.258 | 0.023 |
|  | 11 | 0.697 | 0.031 | -0.011 | 0.044 | -0.056 |  | 8 | -27.256 | 6.520 | 0.012 |
|  | 12 | 0.689 | 0.027 | -0.009 | 0.020 |  | -0.013 | 8 | -27.095 | 6.681 | 0.011 |
|  | 13 | 0.697 | 0.031 | -0.011 | 0.043 | -0.053 | -0.001 | 9 | -25.257 | 8.519 | 0.004 |
